# Supplementary material for: Cloud BioLinux: pre-configured and on-demand bioinformatics computing for the genomics community
Source: BMC Bioinformatics. 2012 Mar 19;13:42. doi: 10.1186/1471-2105-13-42 (PMC3372431; doi:10.1186/1471-2105-13-42)
Supplement: Additional file 1 — Supplementary 1 Cloud BioLinux software documentation in the form of a mini, self-contained website. Users need to download and uncompress the .zip file, and open through a web browser the "index.html" file available on the main directory. (ZIP 1823 kb). [file 1471-2105-13-42-S1.ZIP › Cloud-BioLinux-Package-Documentation/docs/clmformat.html]

Bio-Linux Software Documentation Pages

Back to search form

## clmformat

|  |  |
| --- | --- |
| Name | clmformat |
| Description | **clmformat** has the primary function of displaying cluster results and associated confidence measures in a readable form, by listing clusters in terms of the labels associated with the indices that are used in the mcl matrix. The labels must be stored in a so called *tab* file; see the **-tab** option for more information. |
| Homepage | http://micans.org/mcl |
| Remote Documentation | http://micans.org/mcl/man/distindex.html |

The primary function of **clmformat** is to display cluster results and
associated confidence measures in a readable form, by listing clusters in
terms of the labels associated with the indices that are used in the mcl
matrix.
